# Scoping Review: Psychotherapeutic Interventions in Older Adults With Depression and Anxiety

**DOI:** 10.1192/bjo.2025.10106

**Published:** 2025-06-20

**Authors:** Ellena Businge, Danielle Bilkey, Chris Fox, Louise Allan

**Affiliations:** 1 The University of Exeter, Exeter, United Kingdom; 2 Cornwall Partnership Foundation NHS Trust, Cornwall, United Kingdom

## Abstract

**Aims:** The objective of this scoping review is to understand the extent and type of evidence in relation to psychotherapeutic interventions for older adults with depression and/or anxiety.

**Methods:** Prior to undertaking this scoping review, a preliminary search of the Cochrane Database of Systematic Reviews, JBI Evidence Synthesis, PubMed, CINAHL and American Journal of Psychiatry was conducted and no identical current or underway systematic reviews or scoping reviews on the topic were identified. The terms ‘old age psychiatry’ and ‘psychotherapy’ were used, and results were filtered for reviews and systematic reviews only. The scoping review will be conducted in accordance with the JBI methodology for scoping reviews. An electronic search for articles will be conducted using PubMed, American Journal of Psychiatry, Cambridge Core (BJPsych,) PsychInfo and Open Journal of Psychiatry. The databases will be searched for the following components: Older person’s mental health (using terms geriatric psychiatry, older person’s mental health, old age psychiatry.) AND Psychotherapy (using terms psychotherapy, talking therapies, cognitive behavioural therapy, cognitive analytical therapy, interpersonal therapy, group therapy, dialectical behavioural therapy, mindfulness, self help, psychodynamic therapy, psychoanalytical therapy, brief intervention, motivational interviewing.) AND Depression (using terms depression, loneliness, suicide, low mood) OR anxiety (using terms anxiety, panic disorder, generalised anxiety) using the title and abstract.

Only primary research studies to be included. Once the articles have been retrieved, they will be saved to an Excel spreadsheet and uploaded to Rayyan. The articles will then be checked and any duplicates will be removed. Two reviewers will check the articles by abstract and either include, exclude. The included articles will then be read, and analysed and written up into the scoping review report.

**Results:** Preliminary search: This returned 570 articles: 109 of these were relevant to this topic. The results returned 855. **Results:** 121 were included and 734 excluded. Psychotherapy research is worldwide but the main areas for primary research are North America and Europe. The majority of papers were randomised controlled trials looking at short-form therapy such as randomised controlled trials.

**Conclusion:** This scoping review has provided a foundation for the current evidence base looking at psychotherapeutic interventions for older adults with anxiety and depression. It would be good to do a similar review for older adults with other mental disorders such as mild cognitive impairment. It provides the foundation for researchers to move on to systematic reviews.

